# Concomitant electromagnetic navigation transbronchial microwave ablation of multiple lung nodules is safe, time-saving, and cost-effective

**DOI:** 10.1016/j.xjtc.2023.07.027

**Published:** 2023-08-19

**Authors:** Joyce W.Y. Chan, Rainbow W.H. Lau, Aliss T.C. Chang, Ivan C.H. Siu, Cheuk Man Chu, Tony S.K. Mok, Calvin S.H. Ng

**Affiliations:** a Division of Cardiothoracic Surgery, Department of Surgery, Prince of Wales Hospital, The Chinese University of Hong Kong, Hong Kong SAR, China; b Department of Imaging and Interventional Radiology, Prince of Wales Hospital, The Chinese University of Hong Kong, Hong Kong SAR, China; c State Key Laboratory of Translational Oncology, Department of Clinical Oncology, Prince of Wales Hospital, The Chinese University of Hong Kong, Hong Kong SAR, China

**Keywords:** concomitant ablation, lung ablation, lung cancer, lung metastases, lung preservation strategy, microwave ablation, transbronchial ablation

## Abstract

**Objectives:**

Transbronchial microwave ablation of lung nodules using electromagnetic navigation bronchoscopy is an emerging local therapy for lung oligometastases and multifocal lung cancers as part of a lung-preserving strategy. Concomitant ablation of multiple lung nodules in a single operating session may provide a one-stop solution.

**Methods:**

Between April 2019 and April 2023, 25 patients had 2 or more lung nodules ablated concomitantly in our hybrid operating room. Nodules were proven or highly suspicious of malignancies or metastases. Feasibility and safety were retrospectively reviewed.

**Results:**

A total of 56 nodules in 25 patients received concomitant multi-nodular ablation. The mean age of patients was 60 years, and the reasons for the lung-preserving strategy were multifocal lung cancers (80%) and lung oligometastases (20%). Among those with multifocal disease, 65% had previous major lung resection for lung cancer. Two to 4 nodules were ablated in each session. The mean nodule size was 9.9 mm (range, 5-20 mm), and the mean minimal margin was 5.9 mm. When comparing concomitant nodule ablation with the 103 single-nodule ablations performed in our institute, a mean of 86 minutes of operative time and 131 minutes of anesthetic time were saved. There were no increased complications despite overlapping ablation zones, and the mean hospital stay was 1.23 days. The rate of pneumothorax was 8%, and that of pleural effusion, pain, and fever was 4% respectively.

**Conclusions:**

Concomitant transbronchial microwave ablation of multiple lung nodules is feasible, safe, and associated with reduction in overall anesthetic and operative time. It is an important armamentarium in the contemporary lung-preserving strategy for battling multifocal lung cancers or lung oligometastases.

## Graphical abstract

**Figure:**
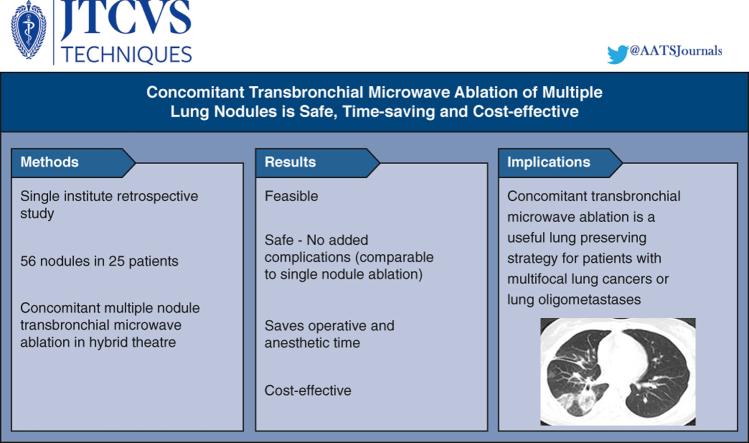

**Figure:**
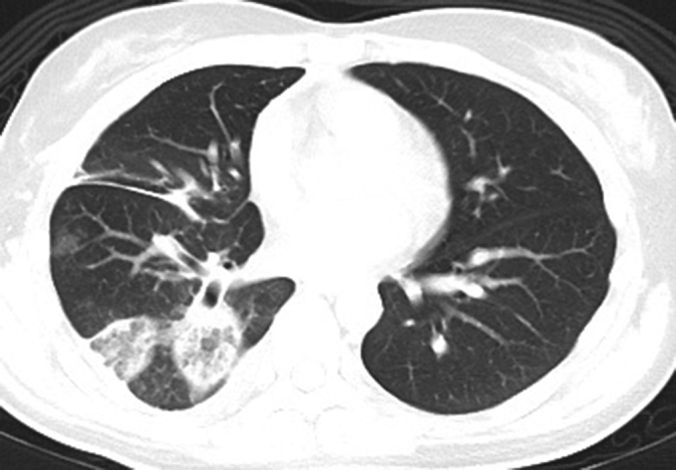
Ablation zones of 2 right lower lobe nodules after concomitant MWA.

**Central Message**

Concomitant transbronchial MWA in the same operative session is feasible, safe, time-saving, and cost-effective as one-stop treatment for multifocal lung cancers and lung oligometastases.

**Perspective**

The increasing prevalence of multifocal lung cancers and lung oligometastases calls for lung-preserving local treatment such as transbronchial MWA. Proof of safety, feasibility, and cost-effectiveness of concomitant multi-nodular ablation are essential for the adoption of this one-stop option to streamline patient care and improve patient satisfaction.

See Discussion on page 273.

Since population-based lung cancer screening has been proven to be cost-effective,^1^^,^^2^ computed tomography (CT) is increasingly being performed each year, leading to an exponential increase in the discovery of incidental lung nodules. Many patients are found to have multifocal lung cancers, and a stage shift toward earlier detection^3^ allows effective treatment and longer survival. In addition, advancement in treatment of other later-stage cancers gives rise to a larger proportion of patients with lung oligometastases. Both of these situations call for local treatment, ranging from sublobar surgical resection, stereotactic body radiation therapy (SBRT),^4^ to percutaneous ablation techniques including radiofrequency ablation (RFA),^5^ microwave ablation (MWA),^6^ and cryotherapy,^7^ which are associated with satisfactory 2-year local control rates between 64% and 70%. The novel technique of combining transbronchial access, offering the potential benefit of fewer pleural-based complications, with MWA, which has been shown to produce larger, faster, and more predictable ablation zones than RFA,^8^ has been developed and used in our institute for 4 years with satisfactory safety and efficacy profile.^9^^,^^10^ Because of the nature of these disease entities, patients are likely to have multiple lung nodules that require local treatment, and coupled with the low complication rate of single-session transbronchial MWA, surgeons at our institute started performing concomitant MWA for multiple lung nodules in the same operative session for indicated patients. In this article, we retrospectively discuss the safety, feasibility, and short-term outcomes of concomitant transbronchial MWA in comparison with a cohort of single ablation cases.

## Materials and Methods

### Trial Design

This study is a single-center retrospective analysis of patients who underwent electromagnetic navigation bronchoscopy (ENB) transbronchial MWA of multifocal lung cancers or lung oligometastases. The study was conducted in compliance with the Declaration of Helsinki and approved by the local Institutional Review Board (the Joint Chinese University of Hong Kong–New Territories East Cluster Clinical Research Ethics Committee, CREC Reference No. 2020.524, May 24, 2020). All study participants gave informed consent for publication of study data.

### Enrollment Criteria

Patients with confirmed or radiologically suspicious T1N0M0 (TNM classification 8th edition) multifocal lung cancers or 5 or less lung metastases with controlled primary malignancy are eligible for consideration of ENB transbronchial MWA if they are unfit for surgical resection or have borderline lung function. Favorable nodule factors include presence of bronchus sign (segmental airway seen leading to lesion), tumors less than 2.5 cm in size, and tumors at least 5 mm away from large blood vessels or sensitive mediastinal structures.

### Concomitant Electromagnetic Navigation Bronchoscopy Transbronchial Microwave Ablation Procedure

**Figure 1:**
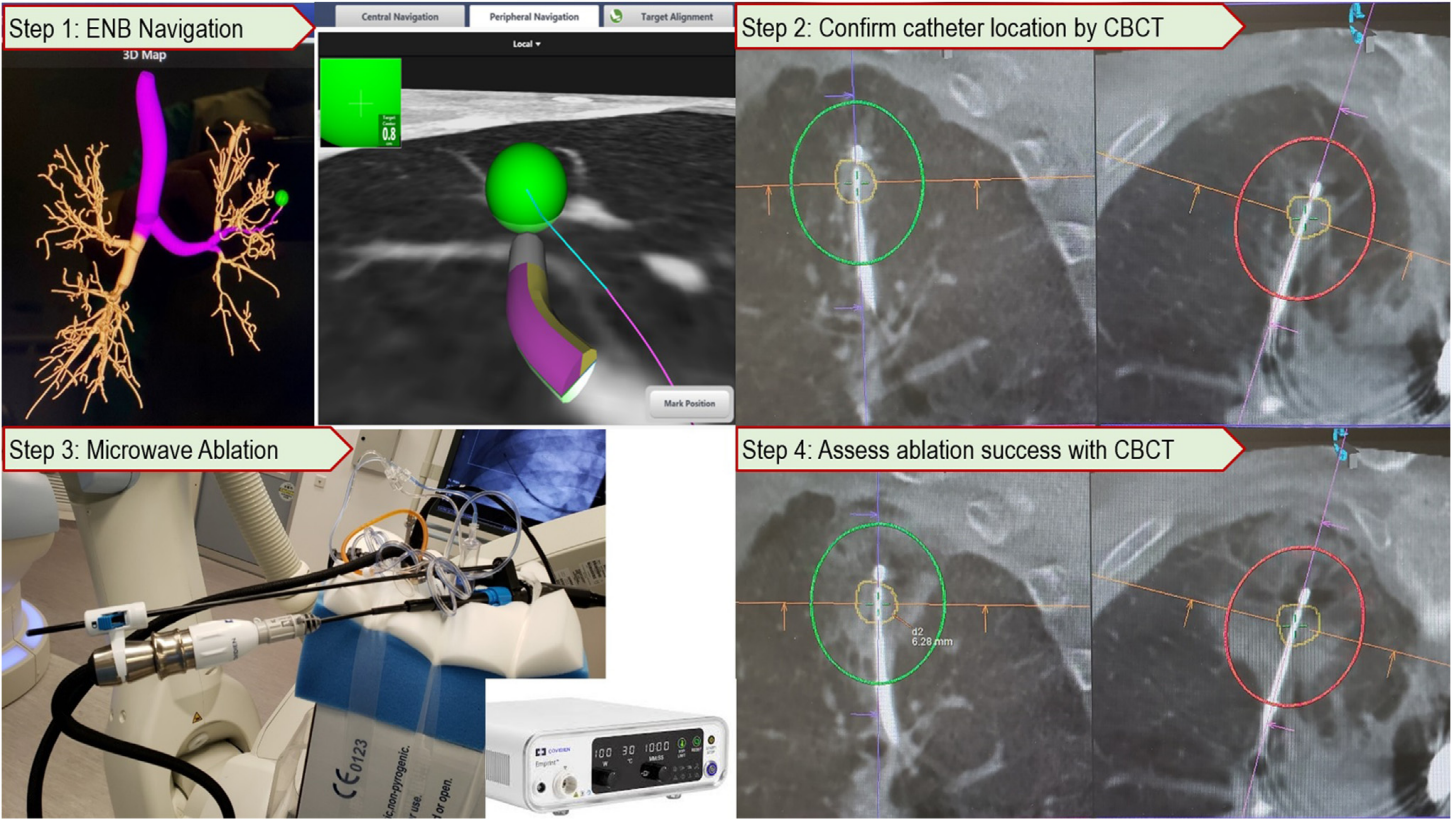
Key procedural steps of transbronchial MWA using ENB are shown. The first step consists of navigation toward the lesion (*green ball*) using ENB guidance. The second step includes confirmation of locatable guide in close proximity to lesion, exchanging it to ablation catheter with or without transbronchial access tools, and final unsheathing of the ablation catheter followed by a preablation CBCT. In this case, the lesion is marked by orange outline, punctured by ablation catheter, and the green and red ovals represent the predicted ablation zones. The third step includes MWA, the level of energy used depending on intended ablation size. The last step includes a postablation CBCT showing the actual ablation zone represented by ground-glass opacities covering the original lesion. *ENB*, Electromagnetic navigation bronchoscopy; *CBCT*, cone beam computed tomography.

All ablations were performed in our hybrid operating room with the help of cone beam CT (CBCT) (Artis Zeego PURE platform, Siemens Healthineers, Erlangen, Germany) and fluoroscopy, both as confirmation of lesion access and for assessment of ablation results. The ENB platform used was SuperDimension Navigation System (Covidien, Plymouth, Minn), and ablation was performed with Emprint Ablation Catheter with Thermosphere technology (Covidien). The procedural steps for single-nodule MWA have been described in our previous publication^9^ (Figure 1). During concomitant nodule ablation, patients were anesthetized similarly, the first nodule was navigated to and ablated, and while analyzing the 10-minute postablation CBCT scan, the bronchoscope was withdrawn temporarily from the endotracheal tube to allow better ventilation, to minimize atelectasis, and to avoid carbon dioxide retention due to prolonged procedure. Subsequent ablation to other lung nodules is only carried out if there are no immediate complications after the first ablation. Technical success was defined by postablation CBCT showing ablation zone covering original lesion. The postoperative course was similar to single-nodule ablation, including chest x-rays on day 0 and day 1 and discharged earliest at 1 day postablation if no complications arise. Fine-cut plain CT scans were arranged as per institute protocol at 1, 3, 6, and 12 months postablation, and then twice per year afterward.

## Results

**Figure 2:**
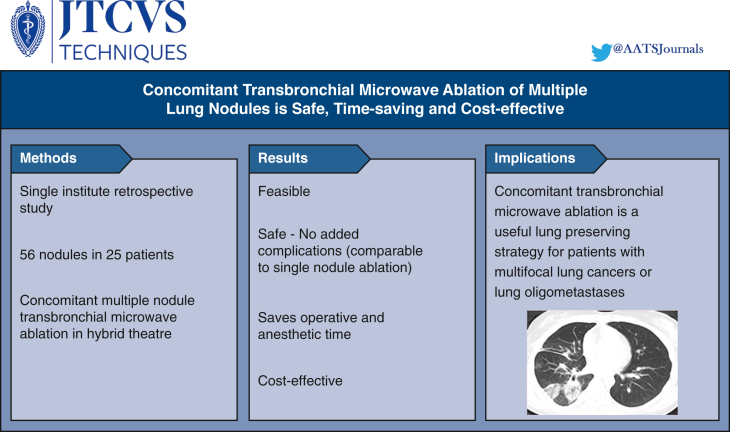
Methods, results, and implications of the present study are outlined.

Between April 2019 and April 2023, 56 nodules in 25 patients (9 male and 16 female) received concomitant transbronchial ENB MWA in our institute. All cases have been discussed in multidisciplinary meeting. The major findings are summarized in Figure 2. The mean age of patients was 60 years (range, 34-79 years). The lesion maximal diameter had a mean of 9.3 mm, ranging from 4 to 20 mm in size. Reasons for lung-preserving strategy in this cohort included lung oligometastases (20%) and radiological or confirmed multifocal lung cancers (80%). Among the latter, 13 of 20 patients have received previous major lung resection, and the rest received lung resection or other local treatment planned for other lesions. The majority of the 25 patients had 2 nodules ablated in the same operative session, 3 patients had 3 nodules ablated, and 2 patients had 4 nodules ablated concomitantly. Seventeen patients had nodules ablated that resided in the same lobe, 7 patients had nodules in different lobes on the same side, and 3 patients had contralateral nodules (Table 1). ENB biopsy was obtained in 2 of the nodules just before ablation, and in 2 other cases ENB ablation was followed by surgical resection of other lung nodules during the same operative session.

**Table 1:** Baseline characteristics of concomitant nodule ablation

	No. of patients	Mean	Range
No. of nodules ablated in the same operative session			
1	103		
2	21		
3	2		
4	2		
Concomitant ablation lesion location			
Same lobe	17		
Different lobes on same side	7		
Different laterality	3		
Patient characteristics (25 patients)			
Age (y)		60	34-79
Gender			
Male	9		
Female	16		
Charlson Comorbidity Index		5.4	
Lesion characteristics (56 lung nodules)			
Lesion size (mm)		9.3	4-20
Lobe			
RUL	16		
RML	2		
RLL	10		
LUL	17		
LLL	11		
Histology			
Lung cancer	7		
Proven metastasis	1		
Not available	48		
Lesion nature			
Pure GGO	23		
Mixed GGO	17		
Solid	16		
Distance to pleura (mm)		13.3	0-34

*GGO*, Ground-glass opacity; *LLL*, left lower lobe; *LUL*, left upper lobe; *RLL*, right lower lobe; *RML*, right middle lobe; *RUL*, right upper lobe.

The results and safety of concomitant ablation were compared with the 103 single-nodule MWAs performed in our institute during the same period (Table 2). The operative time of single-nodule versus double-nodule ablation was 144 ± 11 minutes and 204 ± 39 minutes, respectively, amounting to a mean of 86 minutes difference, or time saved, if the 2 nodules were to be ablated in separate operative sessions. Likewise, the anesthetic time was 183 ± 11 minutes and 235 ± 41 minutes, respectively, estimating 131 minutes saved in time under general anesthesia when the 2 nodules were ablated in the same session. The estimated time saved is the approximate time difference between 2 times that of single-nodule ablation minus that of double-nodule ablation. The mean ablation energy delivered during single- and multiple-nodule ablation was 77569J and 220950J, respectively, the mean radiation dose was 31492 μGym^2^ and 61673 μGym^2^, respectively, and the mean number of CBCTs required was 8.3 and 15.4, respectively (Table 2).

**Table 2:** Comparison between multiple-nodule concomitant ablation and single-nodule ablation

	Single nodule	Double nodule	Time saved
Operative time (min)	144 ± 11	204 ± 39	≈86
Anesthetic time (min)	183 ± 11	235 ± 41	≈131

	Single nodule	≥2 nodules
Ablation energy (J)	77,569 ± 7764	220,950 ± 40,479
Radiation dose (μGym^2^)	31,492 ± 2813	61,673 ± 15,953
No. of CBCTs	8.3 ± 0.5	15.4 ± 2.5

*CBCT*, Cone beam computed tomography.

Of 17 patients who had ablation to the same lobe, 6 of them had overlapping or merged ablation zones on their 1-month postablative CT scan. No complications due to merged ablation zones arose.

**Figure 3:**
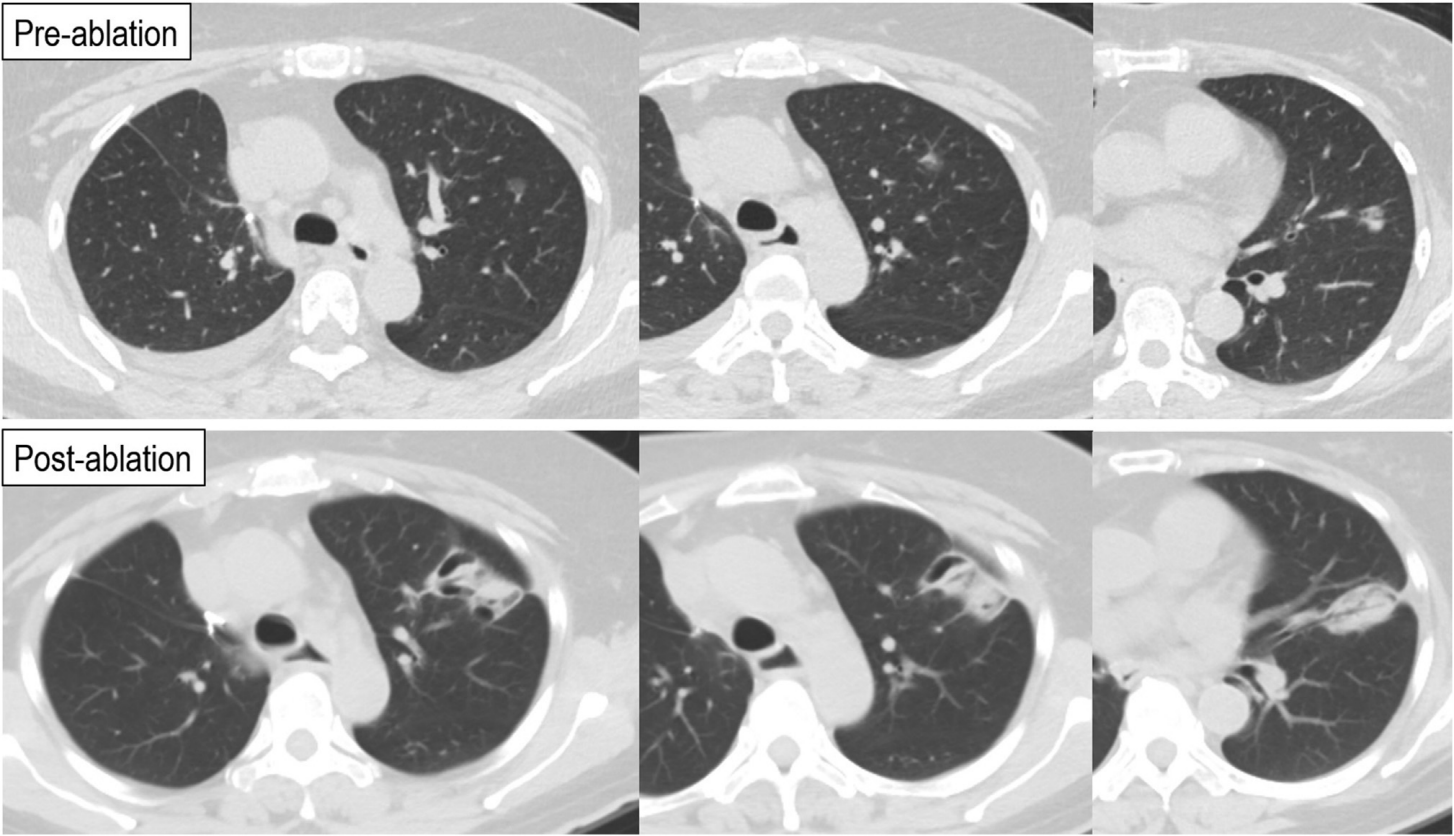
An example of concomitant triple nodule ablation for multifocal lung cancer. The patient had a *right upper* lobectomy and *right lower* lobe wedge for early lung cancer previously. *Top*: preablation CT scans of 3 ground-glass lesions in the *left upper* lobe, which progressed on follow-up. *Bottom*: 1-month postablation CT scans demonstrating 2 closely related and merged ablation zones and another more inferiorly.

Technical success for planned multiple nodule ablation was 100% (Figure 3). The mean minimal margin was 6.1 ± 0.7 mm, which is a conservative measurement given that we do not take into consideration of tissue contraction that can be as much as 40%.^9^ Thirty-five nodules (62.5%) required double or triple ablation to ensure good ablation zone coverage of the nodule, and these include same position reablation, pull-back reablation, and renavigation reablation (bracket ablation). Mean hospital stay for multiple nodule ablation was 1.23 days (range, 1-3 days), in comparison with 1.69 days for single-nodule ablation. Mean postablation day 1 C-reactive protein was 15.6 mg/L for multiple-nodule ablation and 22.7 mg/L for single-nodule ablation, and day 1 white blood cell count was 7.8 × 10ˆ9/L and 9.0 × 10ˆ9/L, respectively. Complications of concomitant ablation included 1 case of pain, 1 case of postablation fever, 1 case of self-limiting pleural effusion not requiring drainage, and 2 cases of perioperative pneumothorax (Table 3). In terms of oncological outcomes of the 56 nodules, there was 1 case of local recurrence and death at a mean follow-up of 406 days.

**Table 3:** Complications of concomitant nodule ablation

Complications of concomitant ablation	No. of patients
Pain	1 (4%)
Fever	1 (4%)
Pleural effusion (not require drainage)	1 (4%)
Pneumothorax	2 (8%)
Pulmonary hemorrhage	0

## Discussion

For multifocal lung cancers and lung oligometastases indicated for local treatment, although surgical resection is the gold standard and provides the most reliable confirmation of treatment success,^11^^,^^12^ many of these patients had unfavorable factors, including inadequate lung function due to previous major lung resection for primary lung cancer, central lung lesion requiring resection of a large amount of lung for a relatively small nodule, higher risk and complexity of redo operation, poor premorbid conditions, and patient reluctance due to prior operative experience. In Asian populations, the proportion of epidermal growth factor receptor–expressing lung tumors is higher than in White populations and commonly present with multiple premalignant or early malignant lesions with different molecular mutation,^13^^,^^14^ sometimes as many as 5 to 10 suspicious ground-glass opacities or mixed lesions in the presenting CT scan. These patients have a higher lifetime risk of lung cancer and likely will need long-term surveillance with CT and multiple treatment sessions for growing lesions. An early lung-preserving consideration is recommended,^15^^,^^16^ acknowledging that these patients will live a reasonably long life if treated appropriately, with diminishing lung reserve after each treatment, and the general approach is to treat the most suspicious lesions first. The dominant lesion may be amenable to lobectomy or sublobar anatomic resection, whereas the remaining growing lesions can be treated with local ablation.^17^ SBRT is a reasonable alternative as local therapy,^18^ although it is limited by the inability to obtain histology during treatment and safety concerns for patients with interstitial lung disease or lesion proximity to critical mediastinal structures such as the heart.^19^ In addition, while treating multiple nodules scattered in different lobes by SBRT, a large portion of lung would have to be irradiated for treatment of small lesions.

Transbronchial MWA is a relatively novel technique, with several authors reporting its safety and feasibility. Zeng and colleagues^20^ reported 7 patients with lung lesions treated by ENB MWA, of whom 1 had mild hemoptysis and pneumonia with good recovery. Bao and colleagues^21^ reported 96 nodules in 65 patients treated with ENB MWA, of whom 57 simultaneously underwent minimally invasive thoracic surgery. The complication rate was low, and there were no local recurrences after a short-term follow-up.

Single-nodule transbronchial ENB MWA has been shown to be safe and efficacious based on our results published 2 years ago.^9^ The mean minimal ablation margin was 5.5 mm, mean hospital stay was 1.73 days, and complication rate was low, including pain (13.3%), pneumothorax (6.7%), postablation reaction (6.7%), pleural effusion (3.3%), and hemoptysis (3.3%). After overcoming the initial learning curve of this novel technique, surgeons in our institute started performing the first concomitant nodule ablation after approximately 35 cases and have been more liberally performing it after the 70th case. In 2 patients, 4 nodules in the same or different lobes have been ablated concomitantly successfully without complication.

There have been concerns regarding multiple-nodule ablation initially. For multiple-nodule percutaneous ablation, the risk of pneumothorax is likely doubled due to multiple pleural punctures.^22^ Even with the transbronchial approach, pneumothorax is a possibility^23^^,^^24^ and ablation to nodules on different sides may theoretically lead to bilateral pneumothorax, leading to ventilatory difficulty if not promptly recognized. Ablation to nodules in the same lobe, on the other hand, may lead to high concentration of energy delivered in a relatively small volume, large and merged ablation zones, the effect of which is uncertain and seldom reported. The possibility of significant postablation reaction with fever, large ablation zone involving pleura causing pneumothorax and bronchopleural fistula,^25^^,^^26^ infection of such large area of necrosis, and significant hemorrhage are among the concerns during our initial experience. Prolonged occupancy of the endotracheal tube by large bore bronchoscope also may increase the risk of carbon dioxide retention and atelectasis during the procedure.

**Figure 4:**
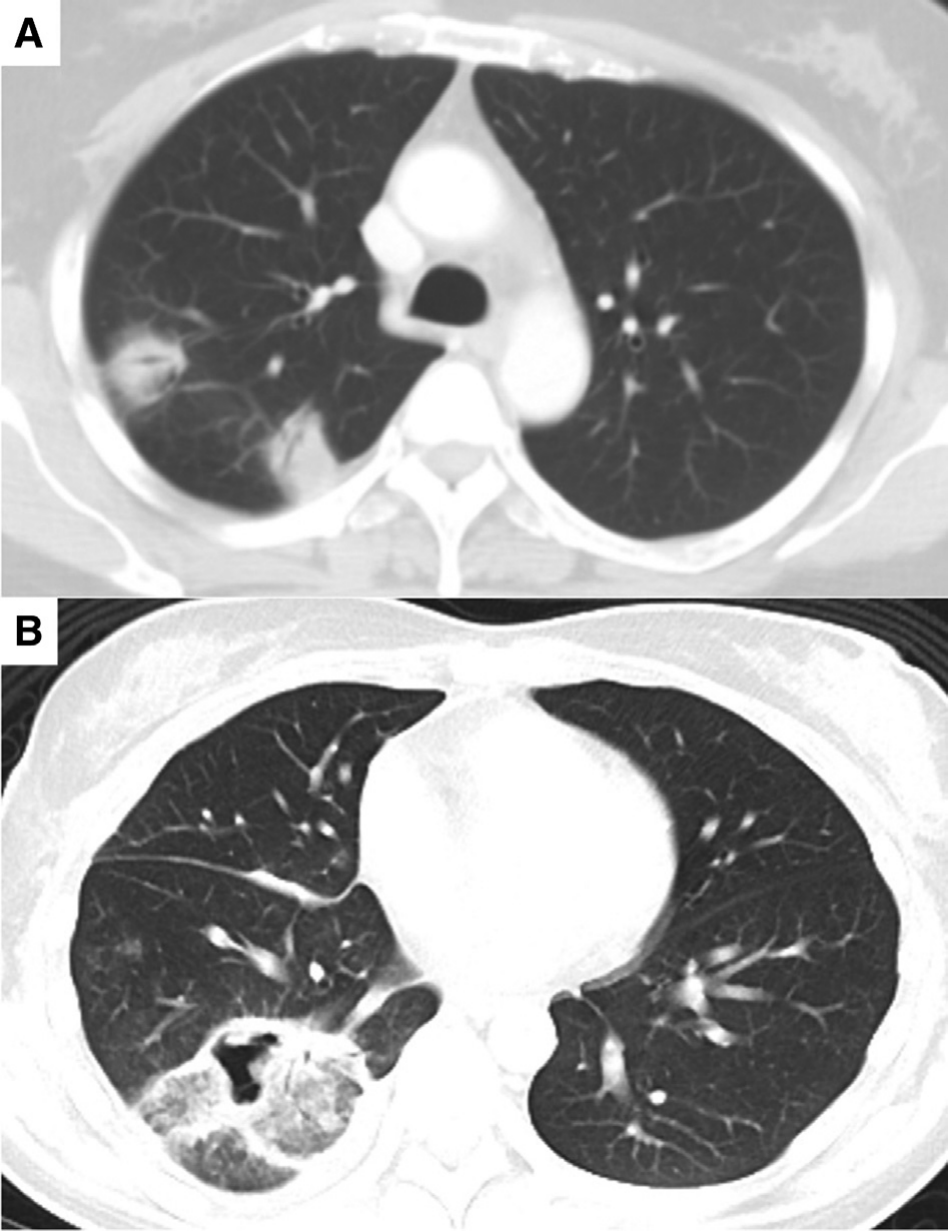
Among patients with multiple concomitant nodule ablation, ablation zones range from small and separate (A) to large and merged ablation zones (B) on 1-month postablation CT. The overlapping ablation zones may develop some cavitation.

Our results show that ENB MWA of multiple nodules in the same operative session is largely safe without added complications. Ablation zones achieved in these 25 patients ranges from small and separate zones to large and merged ablation zones occupying up to half a lobe (Figure 4), whereas the complication rate remains low. There was no statistical difference between postablation day 1 C-reactive protein and white blood cell levels between single or concomitant nodule ablation, which remains low. The mean radiation dose and number of CBCTs required was approximately doubled when comparing single- with multiple-nodule ablation, which is to be expected. Despite at least nearly tripling the ablation energy delivered (mean of 77569J for single nodule and 220950J for multiple nodules), there is no increased report of pain or fever (both ∼4%). There were no major hemorrhages. The 2 cases of pneumothorax did not occur due to merged ablation zone. The first case occurred intraoperatively, due to dilator tool puncturing pleura during exchange and treated with tisseal glue injection via ablation tract, eventually not requiring chest drain insertion. The second pneumothorax occurred several days after ablation with CT showing breached pleura at the ablation zone, requiring endobronchial valve placement,^27^^,^^28^ which was removed 2 months later with healed ablation region. The culprit ablation zone is separate from the other nodule and has been ablated only once with 100W for 10 minutes. The 6 patients with overlapping or merged ablation zones did not experience complications. Although not directly related to the effects of multiple ablation, the pneumothorax rate was 8%, similar to that of single-nodule ablation (6.7%). Because pneumothorax is often the direct complication of ablation to a single nodule, the actual pneumothorax rate based on nodule number is only 3.6% (2 occurrences in 56 nodule ablations), an improvement from our previous early results,^29^ representing heightened cautiousness and refined technique. Unfortunately, there is a significant difference between the distance to pleura in the single- and multiple-nodule cohorts (7.46 vs 13.3 mm, *P* < .05), making direct comparison of pneumothorax rate difficult.

Concomitant nodule ablation benefits from fewer general anesthesia sessions, providing 1-stop treatment for patients with multiple lung malignancies. The estimated time required for preprocedural patient positioning, intubation, bronchoscopic toileting and assessment, and ENB registration and verification was approximately 40 minutes for each case. In the comparison between single- and double-nodule ablation, a mean of 86 minutes of operative time and 131 minutes of anesthetic time was saved due to concomitant ablation instead of separate session ablation. With no added complications, patients are likely to report better overall satisfaction due to reduced sum of hospital stay and doctor visits. Furthermore, the same ablation catheter can be used for concomitant ablation, thus reducing overall cost. Of note, the duration of procedure time depends on the ease of navigation and presence of bronchus sign, which ranges from 15 minutes to 1 hour, whereas the meticulous evaluation of postablation CBCT and subsequent reablations take up the rest of the usual procedure time.

There were 2 cases where resection and ablation were performed in the same operative session. In the first case, the patient had 2 relatively deep and faint left upper lobe nodules that were ablated, subsequently proceeding to a left lower apical segmentectomy for a larger and more solid nodule. In the other case, the patient had 2 deep right lower lobe nodules ablation followed by video-assisted thoracic surgery in the right upper and middle lobe wedges for relatively peripheral lung metastases. The combination of approaches enables maximal lung preservation in patients with multifocal lung cancers.

With increasing experience, surgeons at our institute have developed certain tips and tricks while performing concomitant ablation. After the first nodule ablation, immediate postablation CBCT was performed to grossly assess the adequacy of ablation, and the bronchoscope together with ablation catheter was removed from the endotracheal tube if the ablation zone size is preliminarily satisfactory. Before the 10-minute postablation CBCT and while detailed assessment of ablation zone and determining exact margin was performed, the patient is ventilated without bronchoscope blocking half the lumen, thus effectively removing carbon dioxide in preparation for a prolonged procedure. In addition, navigation to the second lesion can start soon after the first postablation CBCT, because subsequent CBCT for the second lesion was tailored to include the first lesion, from which the final margin of the first lesion can be determined.

Selection of ablation order is also important, because ablating the smaller and fainter nodules in the lung base first will evade the nuisance of atelectasis obscuring the lesion due to the prolonged procedure. Likewise, ablating the nodule with a higher risk last (eg, those adjacent to large blood vessels or extremely close to pleura) will avoid the unfortunate event of significant hemorrhage or pneumothorax occurring after first ablation leading to premature termination of procedure, leaving the second lesion unablated. In this series, there were no cases where complications from the first nodule ablation led to procedure abortion such that the second nodule cannot be ablated. In the unusual circumstance when the first ablation obscured the visualization or targeting of the second lesion in the same lobe, overlay software from the CBCT system was used to reproduce the location of the second nodule for navigation and ablation.

## Conclusions

Concomitant transbronchial ENB MWA of multiple lung nodules in the same operative session is technically feasible and safe without added complications. Patients benefit from fewer general anesthetic sessions, fewer hospital visits, shorter overall operative and anesthetic time, and lower cost. In the era of multifocal lung cancers and lung oligometastases amenable to local therapy, this 1-stop treatment is an important component of lung-preserving strategy.

### Webcast

You can watch a Webcast of this AATS meeting presentation by going to: https://www.aats.org/resources/concomitant-electromagnetic-navigation-transbronchial-microwave-ablation-of-multiple-lung-nodules-is-safe.

**Figure:**
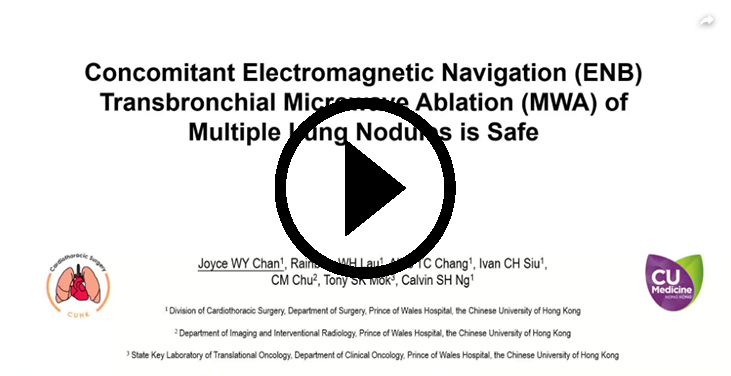

## Conflict of Interest Statement

C.S.H.N. is a consultant for Johnson and Johnson, Medtronic, USA, and Siemens Healthineer. R.W.H.L. is a consultant for Medtronic, USA and Siemens Healthineer. All other authors reported no conflicts of interest.

The *Journal* policy requires editors and reviewers to disclose conflicts of interest and to decline handling or reviewing manuscripts for which they may have a conflict of interest. The editors and reviewers of this article have no conflicts of interest.
